# Visualization of the Persistent Avascular Retina with Ultra-Widefield Green Reflectance Imaging

**DOI:** 10.3390/diagnostics15222873

**Published:** 2025-11-13

**Authors:** Ayşe Cengiz Ünal, Melih Akıdan, Muhammet Kazım Erol

**Affiliations:** 1 Department of Ophthalmology, Antalya Education and Research Hospital, University of Health Sciences, 07100 Antalya, Turkey; muhammetkazimerol@gmail.com; 2 Department of Ophthalmology, Antalya Akseki State Hospital, 07630 Antalya, Turkey; melcihhh@yahoo.com

**Keywords:** retinopathy of prematurity, persistent avascular retina, ultra-widefield fundus imaging, Optos, color reflectance image

## Abstract

**Objectives**: The aim of this study was to determine which color imaging facilitated easier detection of the persistent avascular retina (PAR) in ultra-widefield (UWF) fundus imaging in children undergoing retinopathy of prematurity (ROP). **Methods**: A total of 20 eyes of 10 children aged between 6 and 9 who underwent diagnostic and therapeutic procedures for ROP were included. Fundus images were obtained using Optos confocal scanning laser ophthalmoscopy (cSLO; Optos PLC, Daytona, Dunfermline, UK). The images were divided and recorded into three groups as original imaging (composite), red reflectance imaging, and green reflectance imaging. These images were prepared as a slideshow for 10 ophthalmology specialists and they were surveyed to determine in which color imaging the peripheral avascular area was more easily detected. The results were evaluated. **Results**: The rate of detecting the PAR in green reflectance imaging by the participants included in the study was found to be statistically higher compared to other colors of imaging (composite 0.63 ± 0.09 (0.5–0.8), red 0.12 ± 0.05 (0.05–0.2), and green 0.94 ± 0.06 (0.85–1), *p* < 0.0001). All respondents reported that the boundaries of the peripheral avascular area were more clearly defined in the UWF (Optos PLC, Daytona, Dunfermline, UK) green reflectance imaging. **Conclusions**: Each color imaging used in UWF fundus imaging helps to visualize different layers of the retina. Our study showed that retinal vascular endings appear more distinct due to the lower penetration of the green laser into the choroidal vessels. Based on these findings, we believe that UWF fundus green reflectance imaging is more useful for detecting and monitoring PAR.

## 1. Introduction

Retinopathy of prematurity (ROP) is a disease resulting from abnormal retinal vascularization due to premature birth and low birth weight [1]. Treatment options include laser photocoagulation and anti-vascular endothelial growth factor (VEGF) therapy [2]. In recent years, with the widespread use of anti-VEGF therapy, it has been observed that, in some patients, the vascularization of the peripheral retina is incomplete and the avascular area becomes permanent [3]. This avascular area, which results from the incomplete vascularization after spontaneous resolution of active ROP findings or following anti-VEGF therapy, is defined as persistent avascular retina (PAR) and is among the late outcomes of ROP [4].

Several studies have shown that patients with a history of ROP, whether treated or untreated, may experience tractional or exudative detachments in the late period and these detachments may be associated with PAR [5]. With the recent increase in the proportion of extremely premature infants, it is anticipated that the prevalence of PAR will increase even if ROP treatment is not performed [5].

Compared to the limited 30 to 50-degree field of view of conventional fundus photography, ultra-widefield (UWF) imaging allows visualization of the retina beyond the vortex veins in all quadrants with a single capture, corresponding to a 110 to 220-degree field of view [6]. With UWF imaging, our knowledge of peripheral retinal access and retinal disorders is deepening [7]. This situation has also become extremely important in terms of evaluating peripheral retinal pathologies in children with a history of premature birth.

The Optos confocal scanning laser ophthalmoscope (cSLO; Optos PLC, Daytona, Dunfermline, UK) device can image 200 degrees of the retina in less than 0.5 s without requiring midriasis by providing UWF imaging [8]. Its use in the diagnosis and follow-up of ROP and other peripheral retinal diseases is becoming increasingly widespread [9]. The Optos device uses red (633 nm), green (532 nm), and blue (488 nm) lasers for color imaging and combines these different colored lasers to obtain a retinal image. The green laser provides the best visualization of the sensory retina and RPE, the red laser of the choroid, and the short-wavelength blue laser of the superficial retinal layers [10].

In UWF fundus imaging, various colors of imaging can be used to detect peripheral retinal diseases more easily and quickly [11]. In the present study, we aimed to determine which color imaging facilitated the easier detection of the PAR in UWF imaging in children undergoing ROP.

## 2. Materials and Methods

Approval for this study was obtained from the local ethics committee of the University of Health Sciences, Antalya Training and Research Hospital (approval number: 139-2024; date: 6 June 2024). This cross-sectional, retrospective study was conducted and performed in compliance with the ethical standards set out in the Declaration of Helsinki. Written informed consent was obtained from the parents and/or legal guardians of the patients.

The medical files of patients who were under follow-up for diagnosis and treatment for ROP were retrospectively analyzed between January 2013 and December 2017. A total of 12 children (with a history of prematurity < 34 weeks gestational age and birth weight < 1500 g) aged between 6 and 9 who met the inclusion criteria were scheduled for ophthalmological examination. In our study, 20 eyes of 12 patients who were screened and treated for retinopathy of prematurity were included. Indications for treatment were made according to ICROP [12] criteria in the neonatal period. Laser photocoagulation and anti-VEGF treatment were applied to the eyes requiring treatment. Since laser photocoagulation ablated the avascular area, persistent avascular retina was not observed in these patients. Therefore, eyes treated with laser photocoagulation were not included in our study. Eyes with retinopathy showing spontaneous regression and eyes treated with anti-VEGF for retinopathy were included in the study.

UWF fundus imaging was performed using the Optos device without dilation in 24 eyes included in the study. Images where the nasal and temporal retina were not visualized, images with laser treatment, or eyes with abnormal retinal vasculature except for PAR were excluded from the study. Finally, a total of 20 eyes eligible for evaluating PAR were included. Using the software in the Optos device, images were recorded as original imaging (composite), red reflectance imaging, and green reflectance imaging, resulting in 60 images. Slideshow presentations were prepared by mixing images from different color imaging of the same eye in a randomized order. All images in our study were evaluated by 2 ophthalmologists experienced in ROP (M.K.E. and A.C.Ü.).

Ten ophthalmology specialists were shown images taken with three different colors of imaging on the same monitor. The participants were informed about PAR before the survey. They were given 30 min to evaluate 60 images. The specialists were asked whether there was a PAR area in the images and to mark its boundaries. It was noted how many images each participant detected peripheral avascular retina in for each color of imaging. At the end of the test, the participants were surveyed regarding which color imaging made it easier to detect PAR in the fundus image. The survey results were evaluated by two clinicians experienced in ROP (M.K.E and A.C.Ü).

### Statistical Analysis

Statistical analysis was performed using the SPSS version 23.0 software (IBM Corp., Armonk, NY, USA). Descriptive data were expressed in mean ± standard deviation (SD), median (min–max or Q1–Q3), or number and frequency, where applicable. The normality test was carried out using the Shapiro–Wilk test. The repeated measures analysis of variance (ANOVA) was used to compare the detection rates obtained from specialists in different settings. A *p* value of <0.05 was considered statistically significant.

## 3. Results

**Figure 1:**
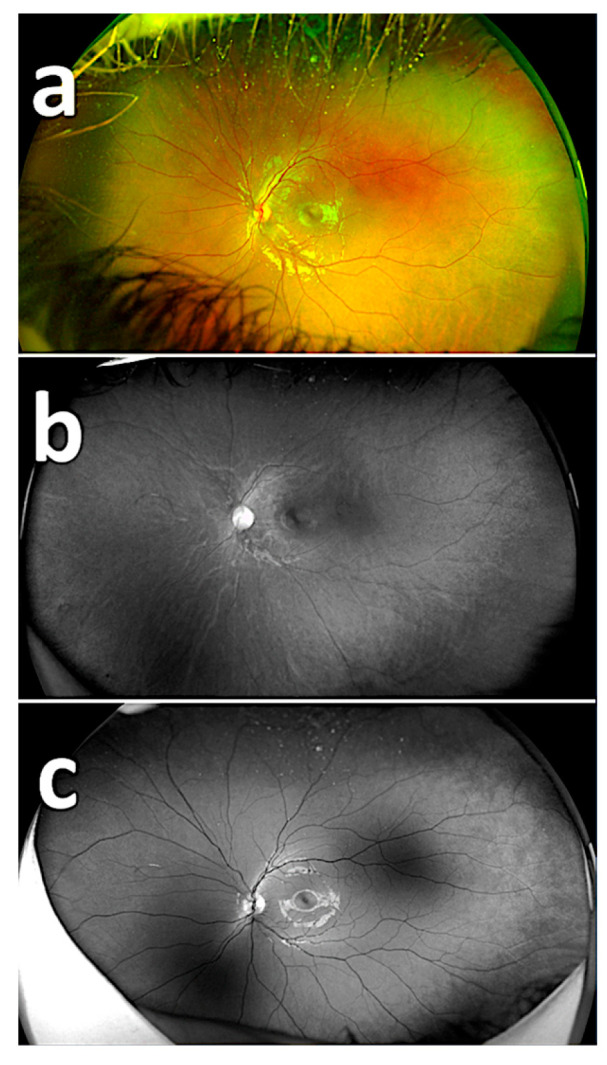
Images from the Optos device. Fundus image using composite imaging (**a**); fundus image using red reflectance imaging (**b**); fundus image using green reflectance imaging (**c**).

The mean age of the patients was 7.7 ± 1.15 (range, 6 to 9) years. The fundus images included in the study were organized using the device’s software as composite imaging (20), red reflectance imaging (20), and green reflectance imaging (20), and the specialists were shown 60 images for detecting PAR (Figure 1). The rate of detecting PAR by the specialists was found to be 12.7/20 in the composite imaging, 2.5/20 in the red reflectance imaging, and 18.7/20 in the green reflectance imaging (Table 1). All respondents reported that PAR could be detected in some images with composite imaging, that detection with the red reflectance imaging was extremely difficult, and detection with the green reflectance imaging was easier due to the clearer visibility of retinal vessels.

**Figure 2:**
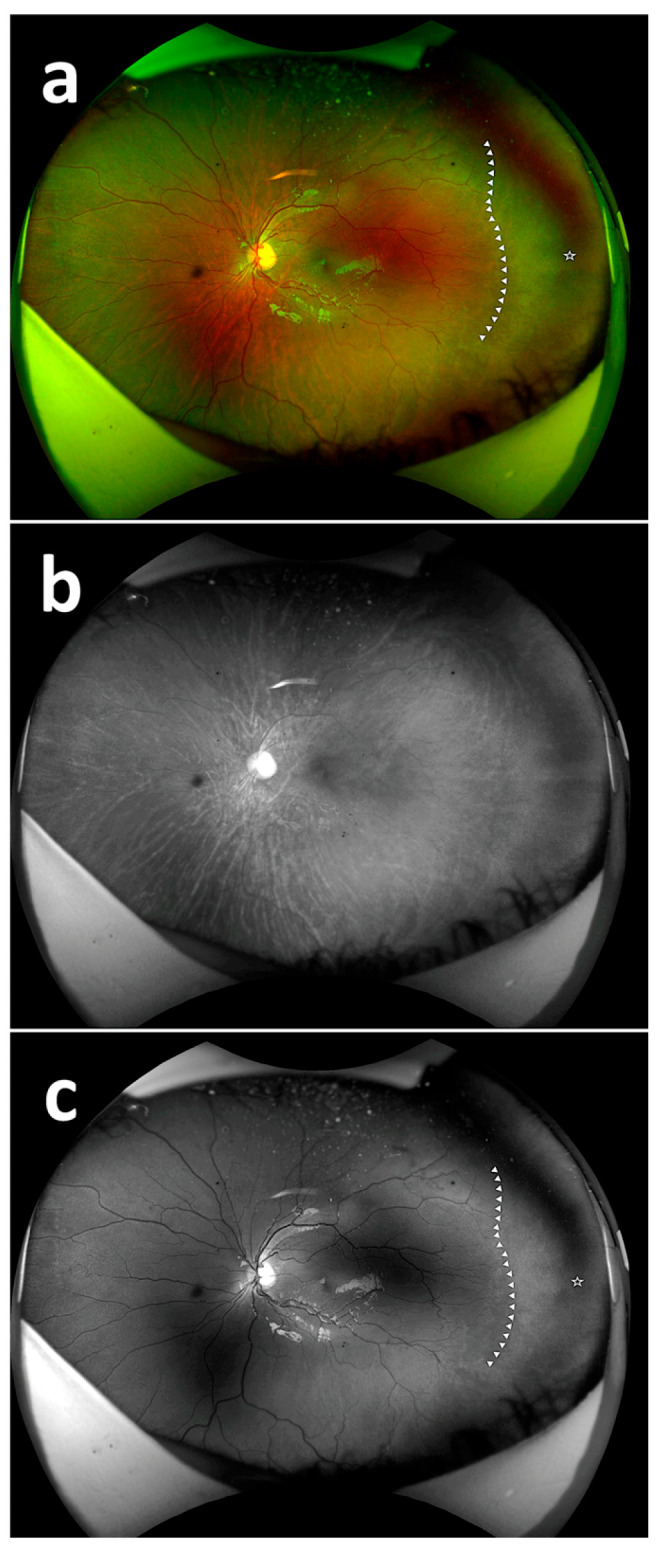
Fundus images with PAR. Fundus image with PAR using composite imaging (arrow: beginning of avascular zone, star: avascular area) (**a**); fundus image with PAR using red reflectance imaging (**b**); fundus image with PAR using green reflectance imaging (arrow: beginning of avascular zone, star: avascular area) (**c**).

**Figure 3:**
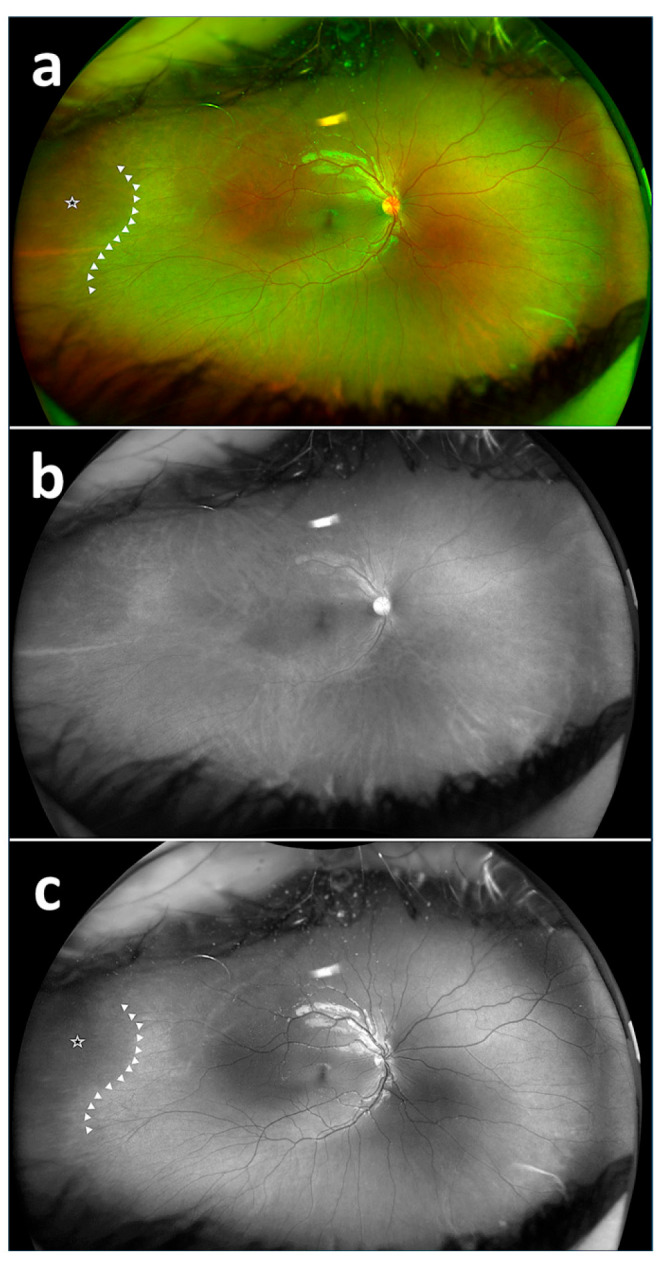
Fundus images with PAR. Fundus image with PAR using composite imaging (arrow: beginning of avascular zone, star: avascular area) (**a**); fundus image with PAR using red reflectance imaging (**b**); fundus image with PAR using green reflectance imaging (arrow: beginning of avascular zone, star: avascular area) (**c**).

**Figure 4:**
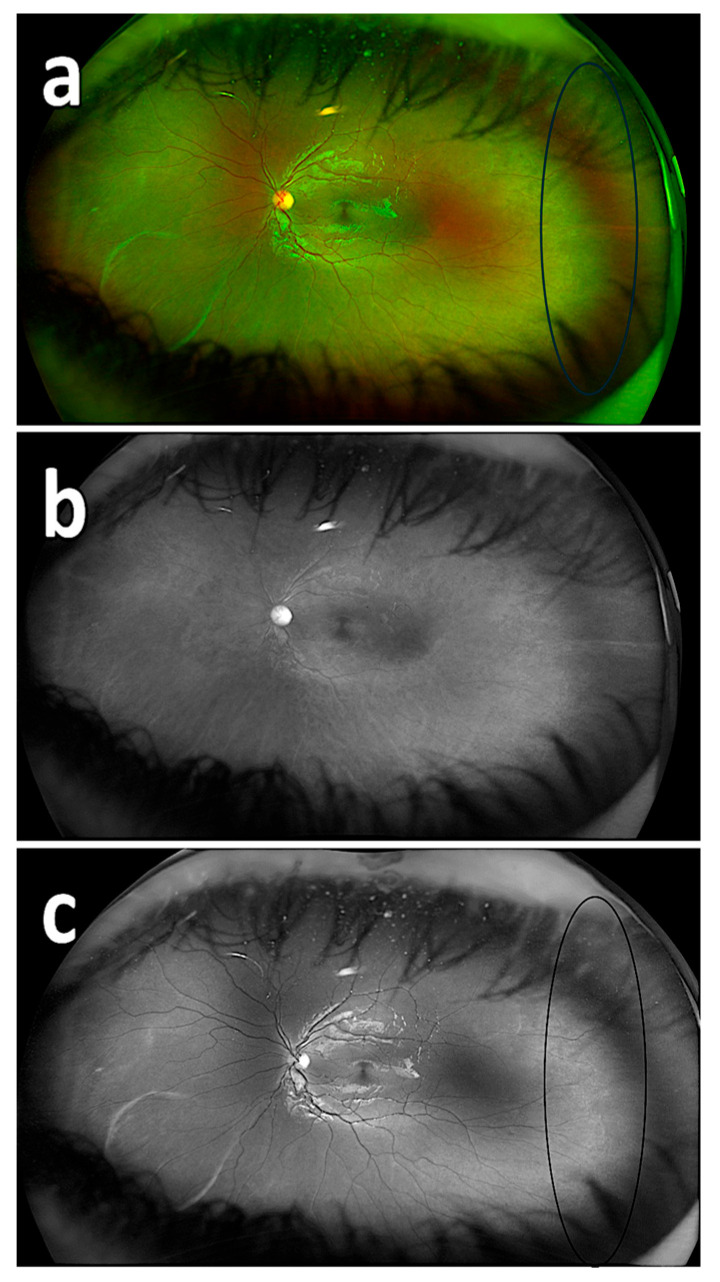
Fundus images with PAR. Fundus image with PAR using composite imaging (ring: beginning of avascular zone) (**a**); fundus image with PAR using red reflectance imaging (**b**); fundus image with PAR using green reflectance imaging (ring: beginning of avascular zone) (**c**).

The rates of detecting PAR areas by the respondents using the composite imaging (a), red reflectance imaging (b), and green reflectance imaging (c) are shown in Table 2. There was a statistically significant difference among the success rates of detecting PAR on composite imaging, red reflectance imaging, and green reflectance imaging based on the 20 images (*p* < 0.05). The success rate of detecting PAR areas with the green reflectance imaging was statistically significantly different and higher compared to both the red reflectance imaging and composite imaging (*p* < 0.001) (Figure 2, Figure 3 and Figure 4). The success rate of detecting PAR areas with the red reflectance imaging was the lowest compared to the composite and green reflectance imaging (*p* < 0.001).

## 4. Discussion

In the present study, we determined which color imaging facilitated the easier detection of the PAR in UWF fundus imaging in children undergoing ROP. We performed wide-angle fundus imaging using the Optos device in patients with a history of ROP and investigated the detectability/ease of detection of these areas using different colors of imaging among specialists. Our study’s results showed that the use of green reflectance imaging significantly improved the detection and ease of detection of PAR areas. To the best of our knowledge, this is the first study to evaluate PAR using UWF fundus imaging with different colors of imaging.

With the recent widespread use of wide-angle imaging, there are researchers who think that color imaging may facilitate diagnosis. It is known that all retinal layers and pathologies become visible especially when the red-free wavelength is absorbed by melanin [13]. There are studies reporting that when this color imaging is used in ROP cases, the distinction between the avascular and vascular areas provides convenience, especially for beginner clinicians [14]. In another study, it was shown that retinal tears were more easily detected by inexperienced ophthalmologists with this color of imaging [11]. In our study, we evaluated the results by asking the participants, because we thought that UWF green reflectance imaging would facilitate the detection of PAR.

In the literature, there is a limited number of studies evaluating not only peripheral retinal diseases, but also other eye segments using imaging along with colored images. In a study, Iqbal compared image recordings taken during gonioscopy with a smartphone under standard halogen light photographs, warm filter, and green filter conditions [15]. The author concluded that the green color imaging provided enhanced tissue visualization for the trabecular meshwork and highlighted that the use of digital technologies and color imaging enables clearer visibility of both contrast and anatomical structures. Sharma et al. compared the use of yellow and green light during indirect ophthalmoscopy, suggesting that green light not only enhanced patient comfort but also facilitated the visualization of retinal tears, nerve fibers, and atrophies [16]. Ahn et al. compared green and red reflectance imaging in wide-angle imaging presentations of patients with chronic Vogt–Koyanagi–Harada disease [17]. Despite the expected depigmentation of the retinal pigmented epithelium (RPE) and choroid due to the chronic process, they reported that, contrary to expectations, green reflectance imaging was beneficial in determining activation. Inoue et al., on the other hand, compared the use of green and red colored imaging in wide-angle imaging of eyes filled with intravitreal gas after rhegmatogenous retinal detachment surgeries [18]. Following evaluation of 20 eyes, they reported that green reflectance imaging was effective for visualizing superior retinal tears and retinal vasculature, whereas red reflectance imaging was effective for choroidal vasculature. Wai et al. applied digital green colored imaging to the UWF fundus imaging of retinal tears in 10 eyes using an Optos device, providing 17 ophthalmology residents with a maximum of 20 s for each image to determine which filter they could see and how quickly [19]. The study results showed that retinal tears were more easily and quickly detected with green colored imaging. Similarly, Moon et al. attempted to detect peripheral retinal tears in 10 patients by capturing UWF fundus images and evaluating which color of imaging allowed for faster detection of retinal tears [11]. Their study, similar to ours, demonstrated that participants detected tears more easily and quickly in images using green color imaging. The authors concluded that such imaging could prevent retinal tears from being overlooked in telemedicine and artificial intelligence applications.

**Figure 5:**
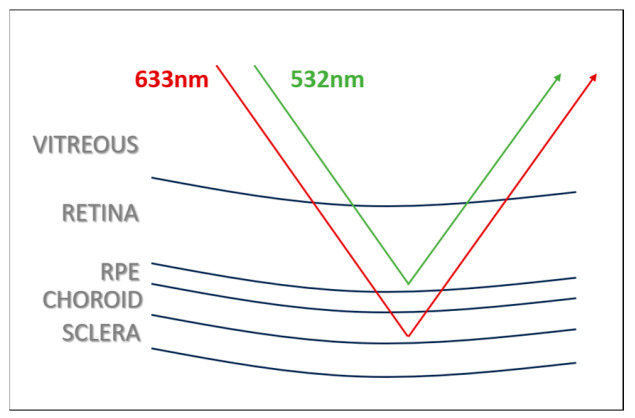
Green and red light penetrance by the laser wavelength.

Retinal imaging is an examination method used in the diagnosis and monitoring of several diseases. Many years ago, retinal imaging started with 20 degrees (Carl Zeiss Company (Oberkochen, Germany) in 1926) and has recently been replaced by wide-angle confocal imaging methods that provide 200 degrees of the retinal field of view (cSLO; Optos PLC, Dunfermline, UK) [20]. Optos, which has been in use since 2000, is a device that uses ellipsoid mirrors to obtain a 200-degree image with a single capture, capable of displaying 82% of the retina [9]. Taking the images by directing the device to the four cardinal positions (up, down, right, and left), retinal imaging can extend up to 220 degrees, covering up to 97% of the retina, although it cannot display the upper and lower retina as wide as the temporal retina [21,22]. Wide-angle imaging of the temporal and nasal retina has popularized the use of the Optos device in peripheral retina diseases. The Optos device utilizes blue (488 nm), green (532 nm), and red (635 nm) laser wavelengths, providing the capabilities of three-channels of color imaging. With its wide imaging capability and the use of different colors of imaging, it has been widely used in the diagnosis and monitoring of vitreoretinal interface, retinal, and choroidal diseases. The device’s software allows for imaging in different colors, enabling the acquisition of single-color, two-color (green–red), and three-color (blue–green–red) images after capture [23,24]. Blue light, with its short wavelength, shows superficial penetration and is particularly effective in highlighting retinal nerve fiber defects and epiretinal membranes. Green light, on the other hand, is effective in visualizing superficial retinal layers, retinal blood vessels, and hemorrhages. Longer wavelengths such as red or near-infrared penetrate deeper, showing choroidal vascularity (Figure 5). As the wavelength increases, larger choroidal veins and multiple vortex ampullae become visible. Therefore, red light, with its longer wavelength, plays an effective role in imaging choroidal lesions such as nevi and tumors [25,26,27]. In the current study, we obtained UWF images using the Optos device and then, we used green and red reflectance imaging to evaluate which color imaging allowed for easier and faster detection of PAR areas. In the composite imaging, both the retina and choroid were visible, whereas in the red reflectance imaging, the choroid was more visible, and in the green reflectance imaging, the retina and vessels were more distinct. Our findings support the claim that PAR areas are more easily and quickly detected with green reflectance imaging, consistent with the literature.

Persistent avascular retina is defined as the cessation of vascularization in the peripheral retinal area, leading to the persistence of non-vascularized areas. The most common cause of avascular peripheral retina is ROP [4]. The third edition of the International Classification of Retinopathy of Prematurity (ICROP) highlights that PAR can occur spontaneously or after anti-VEGF therapy in ROP patients [28]. Multi-center studies have shown that late complications of ROP such as vitreous hemorrhage, retinal detachment, and retinal tears are associated with avascular retina [5,29]. Many researchers consider PAR to be an atypical reactivation of ROP, associated with an increased incidence of early and late-stage retinal detachments [5,30]. However, the majority of these patients are likely to remain asymptomatic and the development of these complications is rare and in a small number of cases. Currently, there is no consensus on the treatment approach for PAR [29]. The approach of detecting PAR and determining which patients require close monitoring, as well as deciding on laser photocoagulation for PAR areas, is further facilitated by wide-angle imaging modalities [31]. The PAR areas can be identified through wide-angle imaging or fundus fluorescein angiography by administering oral or intravenous fluorescein [32,33]. In the present study, we showed that peripheral retina examination could be conducted without fluorescein and dilation, and that PAR could be detected with the aid of color imaging. Additionally, it is known that the gold standard for peripheral retina examination in children requires sedation and indentation [34]. In a recent study, three fundus imaging devices (Topcon color fundus photography, Heidelberg Spectralis MultiColor 55-color montage, and Optos red/green/blue) were compared in 59 eyes with retinal and choroidal pathology. Although no color separation view was used in this study, it is emphasized that peripheral pathologies are better detected with Optos wide-angle imaging, which uses red/green/blue imaging [35]. Our study has indicated that PAR can be detected without the need for sedation and indentation using UWF fundus imaging.

**Figure 6:**
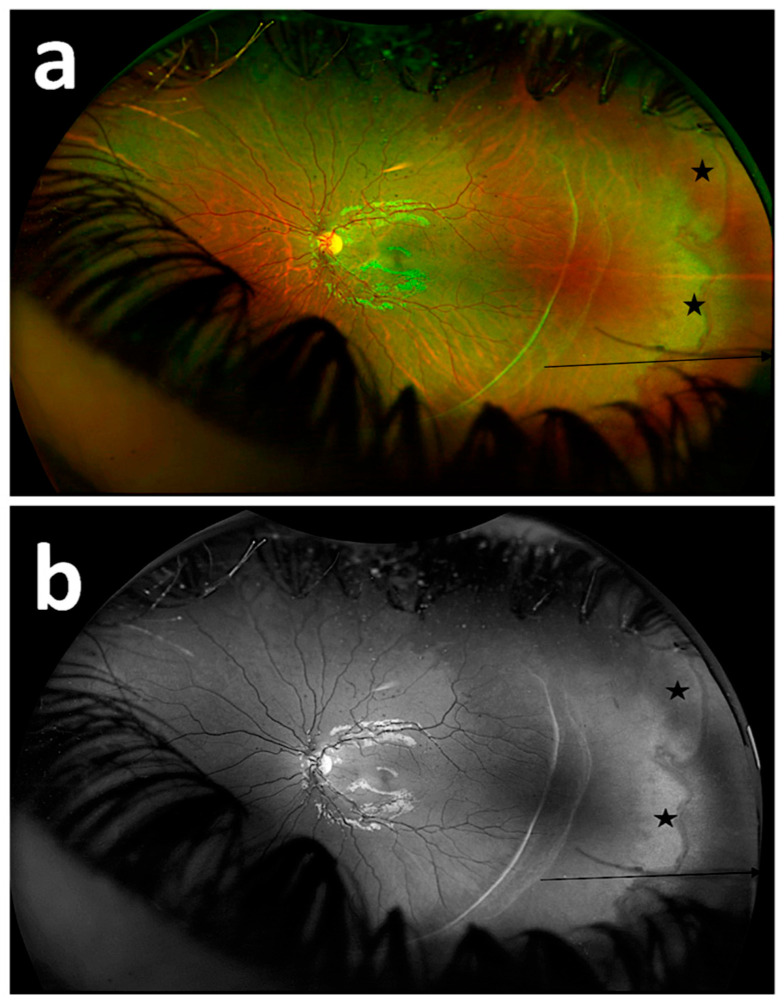
Fundus image of a patient with persistent avascular retina and white without pressure (WWP). Fundus image using composite imaging (**a**); fundus image using green reflectance imaging (**b**). (arrow: persistent avascular area, star: WWP).

Persistent avascular retina can be confused with Familial Exudative Vitreoretinopathy (FEVR) and white without pressure (WWP). FEVR was excluded because the children included in the study had a history of prematurity. WWP most frequently involves the infero-temporal quadrant and supero-temporal quadrants [36]. WWP appears as patchy irregular areas in UWF imaging. It appears more prominent in the green reflectance image and less visible in the red reflectance image. In the green reflectance imaging, it is seen that there is a vein in the WWP area [37,38]. In our study, persistent avascular areas were seen in the temporal quadrant and were not patchy. No retinal vessels were found in these avascular areas (Figure 6).

Nonetheless, there are some limitations to this study. First, the number of participants and patients included in the study is relatively small and future studies with larger sample sizes are needed to obtain more reliable conclusions on this subject. Second, our findings may be subject to chance, as participants’ knowledge levels, interpretations of the images within the designated time, and immediate responses can vary individually. Third, there is a limited number of studies on the use of UWF fundus imaging with color imaging and, to the best of our knowledge, our study is the first to evaluate PAR using color imaging. Therefore, comparisons with previous results regarding PAR cannot be made. Further research is warranted to assess the effectiveness of color imaging and gain new insights into PAR.

## 5. Conclusions

In conclusion, the widespread adoption of UWF fundus imaging provides significant benefits in the management of peripheral retinal diseases such as ROP. We believe that UWF fundus green reflectance imaging, which allows for the visualization of a large portion of the peripheral retina (particularly the temporal quadrant) with a single shot without requiring sedation, indentation, or dilation, will contribute greatly to the diagnosis and monitoring of PAR for clinicians.

## Figures and Tables

**Table 1:** Responses of specialists (the number of PAR views in slides).

No. of Respondents	Composite Imaging (20)	Red Reflectance Imaging (20)	Green Reflectance Imaging (20)
1.	15	3	18
2.	16	2	19
3.	12	1	17
4.	13	4	20
5.	13	3	18
6.	11	2	17
7.	14	1	19
8.	10	4	20
9.	12	3	19
10.	11	2	20

**Table 2:** Comparison of the results of respondents.

Color Imaging	*n*	Mean ± SD (Min–Max)	Median (Q1–Q3)	*p* Value
**Composite**	10	0.63 ± 0.09 (0.5–0.8) A	0.63 (0.55–0.7)	
**Red**	10	0.12 ± 0.05 (0.05–0.2) B	0.13 (0.1–0.15)	
**Green**	10	0.94 ± 0.06 (0.85–1) C	0.95 (0.9–1)	<0.001

Across columns, different uppercase letters indicate statistical differences (*p* < 0.05). *n*: number of participants.

## Data Availability

The data that support the findings of this study are available on request from the corresponding author. The data are not publicly available due to privacy or ethical restrictions.

## References

[1] Hong E.H., Shin Y.U., Cho H. (2022). Retinopathy of prematurity: A review of epidemiology and current treatment strategies. Clin. Exp. Pediatr..

[2] Chung E.J., Kim J.H., Ahn H.S., Koh H.J. (2007). Combination of laser photocoagulation and intravitreal bevacizumab (Avastin) for aggressive zone I retinopathy of prematurity. Graefe’s Arch. Clin. Exp. Ophthalmol..

[3] Tan T.F., Tay S.A., Agarwal-Sinha S., Tan G.S.W., Wu W.C., Tsai A.S.H. (2025). Persistent avascular retina in retinopathy of prematurity. Graefe’s Arch. Clin. Exp. Ophthalmol..

[4] Özdek Ş., Özdemir Zeydanlı E., Baumal C., Hoyek S., Patel N., Berrocal A., Lopez-Cañizares A., Al-Khersan H., Kusaka S., Mano F. (2023). Avascular peripheral retina in infants. Turk. J. Ophthalmol..

[5] Hamad A.E., Moinuddin O., Blair M.P., Schechet S.A., Shapiro M.J., Quiram P.A., Mammo D.A., Berrocal A.M., Prakhunhungsit S., Cernichiaro-Espinosa L.A. (2020). Late-onset retinal findings and complications in untreated retinopathy of prematurity. Ophthalmol. Retin..

[6] Choudhry N., Duker J.S., Freund K.B., Kiss S., Querques G., Rosen R., Sarraf D., Souied E.H., Stanga P.E., Staurenghi G. (2019). Classification and guidelines for widefield imaging. Ophthalmol. Retin..

[7] Kumar V., Surve A., Kumawat D., Takkar B., Azad S., Chawla R., Shroff D., Arora A., Singh R., Venkatesh P. (2021). Ultra-wide field retinal imaging: A wider clinical perspective. Indian J. Ophthalmol..

[8] Hirano T., Imai A., Kasamatsu H., Kakihara S., Toriyama Y., Murata T. (2018). Assessment of diabetic retinopathy using two ultra-wide-field fundus imaging systems, the Clarus^®^ and Optos™ systems. BMC Ophthalmol..

[9] Patel S.N., Shi A., Wibbelsman T.D., Klufas M.A. (2020). Ultra-widefield retinal imaging: An update on recent advances. Ther. Adv. Ophthalmol..

[10] Erginay A. (2024). True-to-life retinal imaging with the new ultrawidefield color RGB modality. Mod. Retin. Digit. Ed..

[11] Moon J.Y., Wai K.M., Patel N.S., Katz R., Dahrouj M., Miller J.B. (2023). Visualization of retinal breaks on ultra-widefield fundus imaging using a digital green filter. Graefe’s Arch. Clin. Exp. Ophthalmol..

[12] International Committee for the Classification of Retinopathy of Prematurity (2005). The International Classification of Retinopathy of Prematurity revisited. Arch. Ophthalmol..

[13] Ducrey N.M., Delori F.C., Gragoudas E.S. (1979). Monochromatic ophthalmoscopy and fundus photography. II. The pathological fundus. Arch. Ophthalmol..

[14] Shakha, Chandra P. (2025). Red-free visualization enhances ease of laser therapy for retinopathy of prematurity. Indian J. Ophthalmol..

[15] Iqbal M.I. (2024). Red-free (green) filter-enhanced gonioscopy with smartphone: A pilot study. Cureus.

[16] Sharma P., Dhami A., Dhami N.B., Dhami G.S. (2022). Comparison of patient satisfaction with red-free (green) versus yellow light using binocular indirect ophthalmoscope for retinal examination. Indian J. Ophthalmol..

[17] Ahn S.E., Kim S.W., Oh J., Huh K. (2013). Ultra-wide-field green (532 nm) and red (633 nm) reflectance imaging of the “sunset glow” fundus in chronic Vogt-Koyanagi-Harada disease. Indian J. Ophthalmol..

[18] Inoue M., Koto T., Hirota K., Hirakata A. (2017). Ultra-widefield fundus imaging in gas-filled eyes after vitrectomy. BMC Ophthalmol..

[19] Wai K.M., Moon J., Dahrouj M., Miller J. (2021). Application of a digital green filter for improved visualization of retinal breaks on ultra-widefield fundus imaging. Investig. Ophthalmol. Vis. Sci..

[20] Ciardella A., Brown D., Agarwal A. (2007). Fundus Fluorescein and Indocyanine Green Angiography: A Textbook and Atlas. New York, Slack Incorporated. Wide Field Imaging.

[21] Choudhry N., Golding J., Manry M.W., Rao R.C. (2016). Ultra-widefield steering-based spectral-domain optical coherence tomography imaging of the retinal periphery. Ophthalmology.

[22] Witmer M.T., Parlitsis G., Patel S., Kiss S. (2013). Comparison of ultra-widefield fluorescein angiography with the Heidelberg Spectralis^®^ noncontact ultra-widefield module versus the Optos^®^ Optomap^®^. Clin. Ophthalmol..

[23] Optos (2021). The Benefits of Optomap. https://www.optos.com/products/the-benefits-of-optomap/.

[24] Stanga P.E., Bravo F.J.V., Reinstein U.I., Stanga S.F.E. (2023). New 200° single-capture color red-green-blue ultra-widefield retinal imaging technology: First clinical experience. Ophthalmic Surg. Lasers Imaging Retin..

[25] Toslak D., Son T., Erol M.K., Kim H., Kim T.H., Chan R.V.P., Yao X. (2020). Portable ultra-widefield fundus camera for multispectral imaging of the retina and choroid. Biomed. Opt. Express.

[26] Biswas S., Khan M.I.A., Hossain M.T., Biswas A., Nakai T., Rohdin J. (2022). Which color channel is better for diagnosing retinal diseases automatically in color fundus photographs?. Life.

[27] Lin T., Shi C., Wu B., Pazo E.E., Shen L. (2023). Vision degrading myodesopsia assessed with optos ultra-widefield scanning laser ophthalmoscope. BMC Ophthalmol..

[28] Chiang M.F., Quinn G.E., Fielder A.R., Ostmo S.R., Paul Chan R.V., Berrocal A., Binenbaum G., Blair M., Peter Campbell J., Capone A. (2021). International classification of retinopathy of prematurity, third edition. Ophthalmology.

[29] Kim J., Kim S.J., Chang Y.S., Park W.S. (2014). Combined intravitreal bevacizumab injection and zone I sparing laser photocoagulation in patients with zone I retinopathy of prematurity. Retina.

[30] Hu J., Blair M.P., Shapiro M.J., Lichtenstein S.J., Galasso J.M., Kapur R. (2012). Reactivation of retinopathy of prematurity after bevacizumab injection. Arch. Ophthalmol..

[31] Robles-Holmes H., Coyner A.S., Campbell J.P., Nudleman E. (2024). Imaging features associated with persistent avascular retina in retinopathy of prematurity. Ophthalmology.

[32] Hanif A.M., Gensure R.H., Scruggs B.A., Anderson J., Chiang M.F., Campbell J.P. (2022). Prevalence of persistent avascular retina in untreated children with a history of retinopathy of prematurity screening. J. Am. Assoc. Pediatr. Ophthalmol. Strabismus.

[33] Yu Y., Wang J., Chen F., Chen W., Jiang N., Xiang D. (2020). Study protocol for prognosis and treatment strategy of peripheral persistent avascular retina after intravitreal anti-VEGF therapy in retinopathy of prematurity. Trials.

[34] Mackenzie P.J., Russell M., Ma P.E., Isbister C.M., Maberley D.A. (2007). Sensitivity and specificity of the optos optomap for detecting peripheral retinal lesions. Retina.

[35] Nagel I.D., Heinke A., Agnihotri A.P., Yassin S., Cheng L., Camp A.S., Scott N.L., Kalaw F.G.P., Borooah S., Bartsch D.G. (2025). Comparison of a Novel Ultra-Widefield Three-Color Scanning Laser Ophthalmoscope to Other Retinal Imaging Modalities in Chorioretinal Lesion Imaging. Transl. Vis. Sci. Technol..

[36] Karlin D.B., Curtin B.J. (1976). Peripheral chorioretinal lesions and axial length of the myopic eye. Am. J. Ophthalmol..

[37] Cheung R., Ly A., Katalinic P., Coroneo M.T., Chang A., Kalloniatis M., Madigan M.C., Nivison-Smith L. (2022). Visualisation of peripheral retinal degenerations and anomalies with ocular imaging. Semin. Ophthalmol..

[38] Orlin A., Fatoo A., Ehrlich J., D’Amico D.J., Chan R.P., Kiss S. (2013). Ultra-widefield fluorescein angiography of white without pressure. Clin. Ophthalmol..

